# Marine n−3 Fatty Acids and Prevention of Cardiovascular Disease: A Novel Analysis of the VITAL Trial Using Win Ratio and Hierarchical Composite Outcomes

**DOI:** 10.3390/nu15194235

**Published:** 2023-09-30

**Authors:** Soshiro Ogata, JoAnn E. Manson, Jae H. Kang, Julie E. Buring, I-Min Lee, Kunihiro Nishimura, Yasuhiko Sakata, Jacqueline Suk Danik, Denise D’Agostino, Samia Mora, Christine M. Albert, Nancy R. Cook

**Affiliations:** 1 Department of Preventive Medicine and Epidemiology, National Cerebral and Cardiovascular Center, Suita 564-8565, Japan; knishimu@ncvc.go.jp; 2 Department of Medicine, Brigham and Women’s Hospital, Harvard Medical School, Harvard T.H. Chan School of Public Health, Boston, MA 02115, USA; jmanson@bwh.harvard.edu (J.E.M.); nhjhk@channing.harvard.edu (J.H.K.); jburing@bwh.harvard.edu (J.E.B.); ilee@bwh.harvard.edu (I.-M.L.); ddagostino@bwh.harvard.edu (D.D.); smora@bwh.harvard.edu (S.M.); christine.albert@cshs.org (C.M.A.); ncook@bwh.harvard.edu (N.R.C.); 3 Department of Cardiovascular Medicine, National Cerebral and Cardiovascular Center, Suita 564-8565, Japan; sakatayk@ncvc.go.jp; 4 Division of Cardiovascular Medicine, Department of Medicine, Massachusetts General Hospital, Harvard Medical School, Boston, MA 02114, USA; jdanik@mgh.harvard.edu; 5 Department of Cardiology, Smidt Heart Institute, Cedars Sinai Medical Center, Los Angeles, CA 90048, USA

**Keywords:** n−3 fatty acid supplementation, cardiovascular disease, coronary heart disease, stroke, win ratio, hierarchical composite outcome

## Abstract

This study aimed to investigate whether n−3 fatty acid supplementation reduced cardiovascular disease (CVD) events in a novel analysis using hierarchical composite CVD outcomes based on win ratio in the VITamin D and OmegA-3 TriaL (VITAL). This was a secondary analysis of our VITAL randomized trial, which assessed the effects of marine n−3 fatty acids (1 g/day) and vitamin D3 on incident CVD and cancer among healthy older adults (*n* = 25,871). The primary analysis estimated win ratios of a composite of major CVD outcomes prioritized as fatal coronary heart disease, other fatal CVD including stroke, non-fatal myocardial infarction (MI), and non-fatal stroke, comparing n−3 fatty acids to placebo. The primary result was a nonsignificant benefit of this supplementation for the prioritized primary CVD outcome (reciprocal win ratio [95% confidence interval]: 0.90 [0.78–1.04]), similar to the 0.92 (0.80–1.06) hazard ratio in our original time-to-first event analysis without outcome prioritization. Its benefits came from reducing MI (0.71 [0.57–0.88]) but not stroke (1.01 [0.80 to 1.28]) components. For the primary CVD outcome, participants with low fish consumption at baseline benefited (0.79 [0.65–0.96]) more than those with high consumption (1.05 [0.85–1.30]). These results are consistent with, but slightly stronger than, those without outcome prioritization.

## 1. Introduction

Effects of marine n−3 fatty acids’ (also called omega−3 polyunsaturated fatty acids) supplementation on cardiovascular disease (CVD) events have been inconsistent in meta-analyses summarizing randomized controlled trials (RCTs) [1,2,3,4,5]. Recent meta-analyses have shown that n−3 fatty acid supplementation may slightly decrease risks of coronary heart disease (CHD) mortality and incident CHD events [2,6,7], but may have little or no benefit for stroke, CVD mortality, total CVD events, or all-cause mortality [2]. In our VITamin D and OmegA-3 TriaL (VITAL), we previously reported that n−3 fatty acid supplementation did not result in a statistically significant reduction in the primary composite CVD outcome (consisting of fatal CVD, and fatal and non-fatal events of myocardial infarction [MI] and stroke) modeled as time-to-first occurrence of any event in the composite with Cox regression analyses, comparing n−3 fatty acids to placebo in all study participants [8]. For the primary composite CVD end point, the hazard ratio was 0.92 (0.80–1.06). However, the VITAL study previously showed a significantly lower incidence of this outcome in the participants whose usual intake of fish was below the median [8]. Also, VITAL previously reported that n−3 fatty acid supplementation significantly reduced a composite CHD end point (consisting of fatal and non-fatal MI, other fatal CHD, and coronary revascularization) but did not reduce the risk of fatal or non-fatal stroke [8]. Those results suggest that differences in usual fish intake at baseline and effects of n–3 fatty acid supplementation on coronary vs. cerebrovascular events should be considered when analyzing composite CVD outcomes.

The inconclusive evidence for the effect of n−3 fatty acid supplementation on CVD events may be due to the fact that most trials modeled composite outcomes as time-to-first event (i.e., ignoring the clinical severity of individual components of composite outcomes) or generally did not account for baseline fish or n−3 intake [2,8]. Individual components of a composite outcome typically have varying impact on human health from non-fatal events to life-threatening ones, though conventional statistical methods typically focus on the time-to-first occurrence of any event in the composite outcome [9,10]. The conventional approaches including Cox proportional hazard models give higher priority to earlier events and may result in non-fatal events being treated as important as fatal ones. The VITAL trial suggested a hypothesis whereby n−3 fatty acid supplementation might have greater health effects in people whose usual n−3 fatty acid intake was relatively low, but meta-analyses have not investigated this hypothesis because most trials did not ascertain baseline n−3 fatty acid intake [2].

The problem of ignoring the differences in the clinical severity of individual components can be solved with a recently introduced statistical method named “win ratio” [9]. The win ratio method can model the priority of individual components of a composite outcome. To the best of our knowledge, no previous RCTs used the win ratio method to evaluate effects of n–3 fatty acid supplementation on composite CVD outcomes with considering differences in the clinical severity of their individual components. Thus, applying this method to the VITAL study, the present study aimed to estimate effects of n−3 fatty acid supplementation on a hierarchical composite major CVD outcome consisting of fatal CHD, other fatal CVD including stroke, non-fatal MI, and non-fatal stroke in this priority order in a general population. As secondary hierarchical composite outcomes, coronary revascularization was added to the above primary composite major CVD outcome, and composites of MI, stroke, and CHD events were examined separately. Additionally, the present study performed the win ratio analyses in subgroups by baseline dietary fish intake to assess whether, as previously seen in VITAL [8], n−3 fatty acid supplementation could confer more cardio-protection in people whose usual n−3 fatty acid intake was relatively low after considering the priority of the components of the composite outcomes.

## 2. Materials and Methods

### 2.1. Study Design and Participants

The present study conducted a secondary analysis using data from the VITAL study, a randomized, double-blind, placebo-controlled trial with a two-by-two factorial design. The detailed study design was reported elsewhere [8,11]. The study aimed to investigate the potential benefits and risks associated with vitamin D3 or n−3 fatty acid supplementation for the primary prevention of CVD and cancer in community-dwelling individuals in the United States. The trial employed a parallel-group 2 × 2 factorial design, randomly assigning participants to one of four intervention groups: vitamin D3 supplementation, n−3 fatty acid supplementation, combined supplementation with both interventions, or placebo. The study recruited eligible individuals from the general population in the United States, specifically targeting men aged 50 years or older and women aged 55 years or older. To be eligible, participants had to be free of any known cardiovascular disease or cancer at the beginning of the study. The planned trial intervention concluded on 31 December 2017, resulting in 25,871 participants with a median follow-up period of 5.3 years (ranging from 3.8 to 6.1 years). A detailed participant flow diagram has been published [8]. As the primary results of the VITAL study, the n−3 fatty acid supplementation, compared to the placebo, did not result in a statistically significant reduction in the primary composite CVD outcome modeled as time-to-first occurrence of any event in the composite with Cox regression analyses in all study participants [8]. However, a significantly lower incidence of this outcome in the participants whose usual intake of fish was below the median was observed in the VITAL study [8].

### 2.2. Intervention and Its Control

Participants were randomized to receive n−3 fatty acids, vitamin D, both active agents, or both placebos between November 2011 and March 2014. Participants assigned to the n−3 fatty acid supplementation group were provided with a daily dose of 1 g of n−3 fatty acids, administered in the form of a fish-oil capsule. The fish-oil capsules contained 840 mg of n−3 fatty acids, including 460 mg of eicosatetraenoic acids (EPA) and 380 mg of docosahexaenoic acids (DHA). The dose of n−3 fatty acids chosen was based on the one recommended by the American Heart Association for CVD protection and reported to be beneficial in a secondary prevention population [12,13]. Participants assigned to the vitamin D3 supplementation group received a daily dose of 2000 IU of vitamin D3. Participants in the combination group received both the vitamin D3 and n−3 fatty acid supplements. Placebo groups received matching placebos corresponding to each intervention (olive oil without any marine n–3 fatty acids). Additionally, the VITAL study provided the participants no dietary counseling or modification except the intervention pills or their placebos. Note that participants were excluded if they took personal fish-oil supplements at baseline, and all participants were advised to forego personal use of fish-oil supplements during the trial. The interventions were administered orally, and participants were instructed to take their assigned supplements or placebos once daily.

### 2.3. Data Collection

The VITAL study collected baseline data on clinical and lifestyle risk factors with questionnaires prior to randomization. These questionnaires also included a dietary assessment section to capture self-reported information on fish consumption and other dietary habits. Follow-up questionnaires were administered annually to monitor adherence to the interventions, identify potential side effects, track the development of major illnesses, and obtain updates on risk factors.

### 2.4. Ethical Considerations

The study received approval from the institutional review board of Partners HealthCare-Brigham and Women’s Hospital. All participants provided written informed consent prior to their enrollment in the trial. The trial agents obtained Investigational New Drug approval from the United States Food and Drug Administration (FDA).

### 2.5. Outcome Assessments

Our proposed primary hierarchical composite outcome was a composite of major CVD events consisting of fatal CHD, other fatal CVD including stroke, non-fatal MI, and non-fatal stroke in this priority modeled with the win ratio method. A secondary hierarchical composite outcome included the above major CVD event composite as well as non-fatal coronary revascularization procedures, specifically, percutaneous coronary intervention (PCI) or coronary artery bypass grafting (CABG), with the lowest priority. As sensitivity analyses, we changed the priority between non-fatal MI and non-fatal stroke because non-fatal stroke could potentially lead to more severe functional decline than non-fatal MI events.

As additional secondary hierarchical composite outcomes, the present analyses considered the components of the above primary and secondary composite outcomes, namely (1) fatal MI and non-fatal MI, (2) fatal stroke and non-fatal stroke, and (3) fatal CHD, non-fatal MI, and non-fatal coronary revascularization procedures in this priority, separately, modeled with the win ratio method. To ascertain the occurrence of the specified end points, the medical records of participants who experienced any of the end points were meticulously reviewed by an independent committee of physicians who were unaware of the trial-group assignments. The confirmation of myocardial infarction and stroke cases relied on established diagnostic criteria [14,15]. Further information regarding the confirmation process for the end points can be found in previous publications and their Supplementary Appendices [8,11].

### 2.6. Statistical Analysis

To investigate the effects of the n−3 fatty acids on the hierarchical composite outcomes, the present analyses used the win ratio method with the intention-to-treat principle. In this method, every patient on the n−3 fatty acids was compared with every patient on the placebo (i.e., comparing every possible patient-to-patient pair), considering a shared follow-up time that was determined as the minimum of their individual follow-up durations. To determine “winners” and “losers” [9], pairs were categorized based on whether participants randomized to placebo experienced an outcome before those randomized to n−3 fatty acids during the follow-up period. In cases where both participants in a pair completed or exited the study without a fatal event (i.e., fatal CHD or other fatal CVD including stroke), their classification was determined with the occurrence of any non-fatal events (i.e., non-fatal MI or non-fatal stroke) in this priority for the primary hierarchical composite outcome. Ties occurred when it was not possible to determine whether a pair was a winner or a loser. The win ratio was calculated by dividing the total number of winner pairs by the total number of loser pairs. Win ratios were inverted to be easily compared with hazard ratios previously reported; thus, the reciprocal win ratios less than 1 indicate benefit of n−3 fatty acids. We estimated reciprocal win ratios using the R package “WinRatio” [16].

We also conducted subgroup analyses with baseline dietary fish intake (<1.5 servings/week or ≥1.5 servings/week, which was the median intake frequency) based on our previous analyses suggesting that baseline quantity of dietary fish intake can modify the effect of the n−3 fatty acid intervention on CVD incidence [8]. The present secondary analysis can be considered as an exploratory analysis using the win ratio method, a novel method. Multiple hypothesis testing was not controlled, and no formal adjustment was applied to the *p*-values or confidence intervals.

## 3. Results

### 3.1. Baseline Characteristics of the VITAL Participants

The baseline characteristics of participants by the n−3 fatty acids and the placebo groups are summarized in Table 1. Of the 25,871 participants, 51% were women. The mean (standard deviation) age was 67.1 (7.1) years at baseline. The median (interquartile range) baseline dietary fish intake was 1.5 (0.9 to 2.5) servings/week. The numbers of incident cases for each component of the hierarchical composite outcomes are summarized in Supplementary Table S1.

### 3.2. The Primary and Secondary Hierarchical Composite Outcomes in All Participants

**Figure 1:**
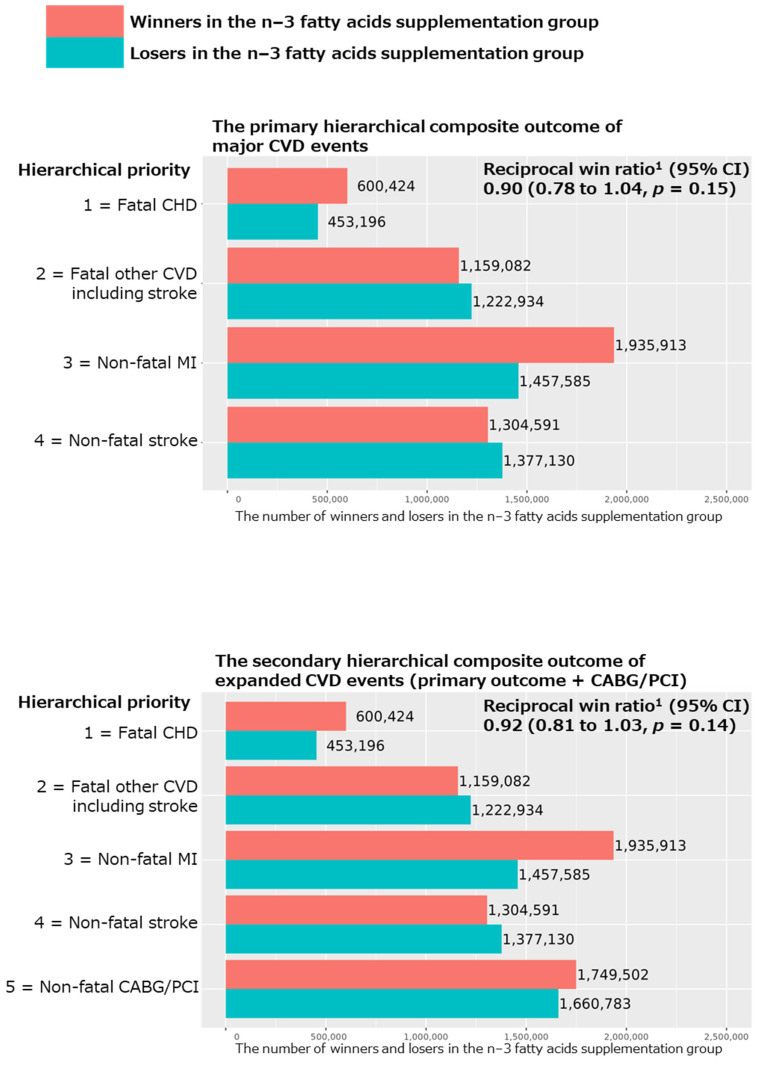
Results of win ratio analyses for the primary and secondary hierarchical composite outcomes comparing n−3 fatty acid group and its placebo group in all participants of the VITAL study. Abbreviations: CVD, cardiovascular disease; CHD, coronary heart disease; MI, myocardial infarction; CABG, coronary artery bypass grafting; PCI, percutaneous coronary intervention; CI, confidence interval. ^1^ Reciprocal win ratio < 1 means beneficial effect of n−3 fatty acid supplementation on the composite outcome.

The win ratio results of the primary hierarchical composite outcome of major CVD events are shown in Figure 1. The total numbers of winners and losers were 5,000,010 and 4,510,845, respectively, in the n−3 fatty acid vs. placebo groups, resulting in slightly but not significantly reduced reciprocal win ratios (95% CI) of 0.90 (0.78 to 1.04; *p* = 0.15) in all participants. The total number of wins was principally influenced by fatal CHD and non-fatal MI events (50.7%).

**Figure 2:**
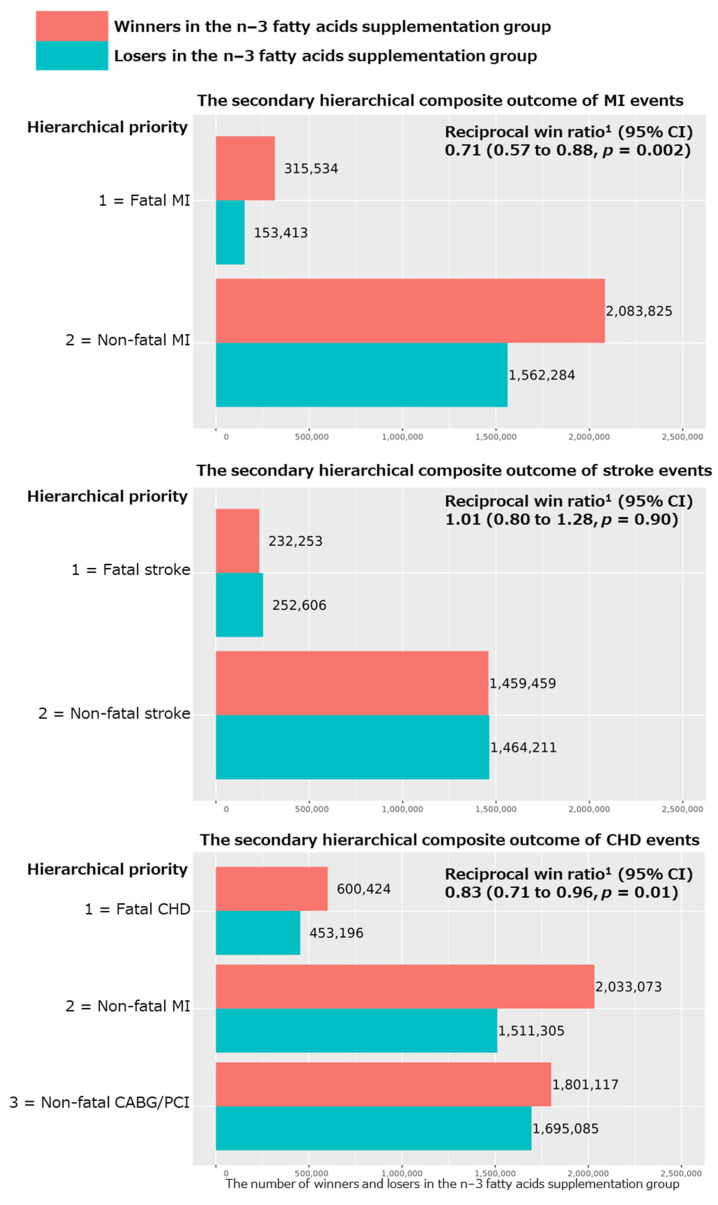
Results of win ratio analyses for the secondary hierarchical composite outcomes of MI, stroke, and CHD events comparing n−3 fatty acid group and its placebo group in all participants of the VITAL study. Abbreviations: CVD, cardiovascular disease; CHD, coronary heart disease; MI, myocardial infarction; CABG, coronary artery bypass grafting; PCI, percutaneous coronary intervention; CI, confidence interval. ^1^ Reciprocal win ratio < 1 means beneficial effect of n−3 fatty acid supplementation on the composite outcome.

For the secondary hierarchical composite outcomes, we added coronary revascularization (CAGB or PCI) to the above major CVD event composite, as well as examined the hierarchical composite outcomes for MI, stroke, and CHD events separately. We obtained nonsignificant reciprocal win ratios (95% CI) of 0.92 (0.81 to 1.03; *p* = 0.14 in Figure 1) and 1.01 (0.80 to 1.28; *p* = 0.90 in Figure 2), respectively, for the hierarchical composite outcomes of expanded CVD (i.e., the primary outcome plus CABG/PCI) and stroke events. However, we obtained significant reciprocal win ratios (95% CI) of 0.71 (0.57 to 0.88; *p* = 0.002) and 0.83 (0.71 to 0.96; *p* = 0.01), respectively, for the hierarchical composite outcomes of MI and CHD events (Figure 2), showing significant benefits of the n−3 fatty acid supplementation for these two outcomes.

### 3.3. The Primary and Secondary Hierarchical Composite Outcomes in Subgroups with Low and High Fish Intake

**Figure 3:**
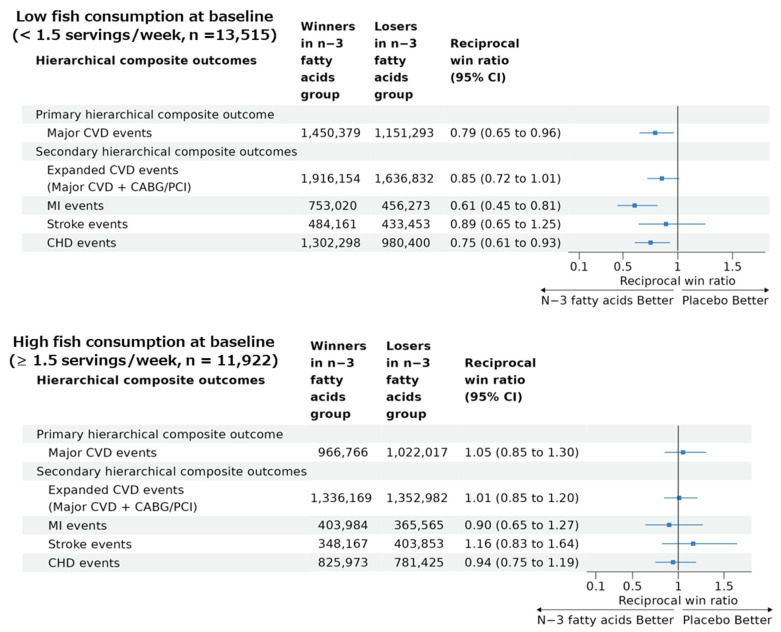
Results of win ratio analyses for the primary and secondary hierarchical composite outcomes comparing n−3 fatty acid group and its placebo group in participants with low and high baseline fish consumption in the VITAL study. Abbreviations: CVD, cardiovascular disease; CHD, coronary heart disease; MI, myocardial infarction; CABG, coronary artery bypass grafting; PCI, percutaneous coronary intervention; CI, confidence interval. Reciprocal win ratio < 1 means beneficial effect of n−3 fatty acid supplementation on the composite outcome.

A significant benefit of the n−3 fatty acid supplementation on the primary hierarchical composite outcome was obtained in the subgroup of participants with lower dietary fish intake (reciprocal win ratio [95% CI] of 0.79 [0.65 to 0.96]), from which the total number of wins and losses was 1,450,379 and 1,151,293, respectively, in the n−3 fatty acid vs. placebo groups (Figure 3). On the other hand, in the participants with higher dietary fish intake (Figure 3), the total number of wins and losses was 966,766 and 1,022,017, respectively, in the n−3 fatty acid vs. placebo groups, resulting in a nonsignificant reciprocal win ratio (95% CI) of 1.05 (0.85 to 1.30). A percentile *p*-value for a difference in the two win ratios was 0.017 based on bootstrapping with 1000 resampling iterations.

Reciprocal win ratios (95% CI) for the four secondary hierarchical composite outcomes in the participants with low and high fish intake at the baseline are summarized in Figure 3. The participants with low fish intake tended to have benefits from n−3 fatty acid supplementation for the four secondary hierarchical composite outcomes with statistically significant reciprocal win ratios for MI (0.61 [0.45 to 0.81]) and CHD (0.75 [0.61 to 0.93]), but not for expanded CVD (0.85 [0.72 to 1.01]) or stroke (0.89 [0.65 to 1.25]) events. However, the participants with high fish intake had no significant benefits of the n−3 fatty acid supplementation on any of the four secondary outcomes. Based on bootstrapping with 1000 resampling iterations, percentile *p*-values for a difference in the two win ratios were 0.08 for expanded CVD, 0.03 for MI, 0.12 for stroke, and 0.07 for CHD hierarchical composite outcomes.

### 3.4. Sensitivity Analyses

As sensitivity analyses, we prioritized non-fatal stroke over non-fatal MI for the primary and secondary hierarchical composite outcomes of major CVD and expanded CVD. The results are similar to the main analyses (Supplementary Tables S2 and S3).

## 4. Discussion

In the present secondary analysis of the n−3 supplementation arm of the VITAL trial, the present study used a novel win ratio method, which can adequately model the priority of individual components of composite outcomes in the RCT. Marine n−3 fatty acid supplementation of 1 g/day had suggestive but statistically nonsignificant benefits on the primary hierarchical composite major CVD outcome in the overall study population; however, n−3 fatty acid supplementation had significant benefits in the participants with lower dietary fish intake (<1.5 servings/week). The benefit of the n−3 fatty acid supplementation shown with the win ratio method is aligned to the primary findings of VITAL, which included 25,871 participants and had a median follow up of 5.3 years, demonstrating that the principal CVD benefits came from reducing the hierarchical composite CHD outcome, but not from reducing the hierarchical composite stroke outcome. Although our previous publication of the main results of the VITAL study did not consider the priority of the individual components of the composite outcome [8], the present secondary analysis was able to consider this, leading to a reduced number of statistical tests and more robust results.

Benefits of the marine n−3 fatty acid supplementation on CHD events were more apparent in VITAL participants with lower dietary intake of fish than those with higher intake, congruent with the main VITAL trial results [8] and with previous meta-analyses including mainly observational cohort studies [17,18,19]. The meta-analyses showed curvilinear associations of usual intake of fish and n−3 fatty acids with CVD and CHD mortality [17,18,19]. Relative risks of the outcomes markedly decreased until approximately a 250 mg/d usual intake of n−3 fatty acids and slightly decreased until 1000 mg/d, but did not apparently decrease with further intake [17,18]. Thus, the benefit of the n−3 fatty acid supplementation on CHD was apparent in people whose usual intake of n−3 fatty acids was relatively low, confirming previous VITAL findings. Authors of the recent meta-analysis had hypothesized that n−3 fatty acid supplementation might have greater health effects in people whose usual n−3 fatty acid intake was relatively low, but they did not test this hypothesis because they were unable to ascertain baseline n−3 fatty acid intake in most trials [2].

In the present analyses, the benefit of the n−3 fatty acid supplementation on CVD events was principally influenced by CHD events rather than stroke events; these results are similar to the main VITAL analyses and recent previous meta-analysis of RCTs [2]. In our analyses, the n−3 fatty acid supplementation showed significant protection against the hierarchical composite CHD and MI outcomes (reciprocal win ratio of 0.83 [95% CI: 0.71 to 0.96] and 0.71 [95% CI: 0.57 to 0.88]), but not the hierarchical composite stroke outcome (reciprocal win ratio of 1.01 [0.80 to 1.28]) or the major CVD outcome (reciprocal win ratio of 0.90 [0.78 to 1.04]). The recent meta-analysis found that increasing n−3 fatty acid intake may slightly reduce the risk of CHD events (relative risk [RR] of 0.91 [95% CI: 0.85 to 0.97]), but may make little or no difference to risk of stroke events (RR of 1.02 [0.94 to 1.12]) and CVD events (RR of 0.96 [0.92 to 1.01]) [2]. Another previous meta-analysis of RCTs showed that n−3 fatty acid supplementation at ≤2 capsules/d corresponding to 1.68–2.52 g/d did not decrease stroke events [4]. However, in this meta-analysis, n−3 fatty acid supplementation at ≥3 capsules/d corresponding to >2.52 g/d versus placebo significantly reduced stroke events (RR of 0.74 [0.57 to 0.95]). Note that the number of papers reporting dose–response effects of n−3 fatty acid supplementation on stroke was limited; thus, further research is needed [4]. The inconsistent results may be attributed to factors like the different underlying causes of stroke including ischemic and hemorrhagic strokes. The hypothesis that n−3 fatty acid supplementation provides protection against coronary events can be scientifically reasonable. A meta-analysis including RCTs and prospective cohort studies supports various possible mechanisms between n−3 fatty acids and CHD, such as lowering triglyceride levels and blood pressure, slowing heart rate, reducing thrombosis, and reducing susceptibility to ventricular cardiac arrhythmias associated with an increase in n−3 fatty acid intake [18]. Further research is required to reveal the different effects of the n−3 fatty acid supplementation on CHD and stroke events.

Our trial has several notable strengths, including representation of the general population encompassing diverse racial, ethnic, and geographic backgrounds. Additionally, the present study used the new statistical method, win ratio analyses considering priority of individual components of a composite outcome, which allowed us to handle composite outcomes more appropriately than conventional methods including Cox proportional hazard models. Furthermore, the VITAL study achieved high rates of participant follow up and adherence to the present questionnaires and the prescribed study pill regimen. The average response rate to the questionnaire was 93.1%, and participants in both the n−3 group and the placebo group reported an average adherence rate to the trial regimen (defined as taking at least two-thirds of the trial capsules) of 81.6% and 81.5%, respectively during the 5-year follow-up period. Throughout the follow up, personal use of fish-oil supplements from outside sources remained below 3.5% in each group.

This trial had several limitations. The duration of the trial intervention, with a median of 5.3 years, could be considered moderate. Moreover, due to the utilization of a single dose level of n−3 fatty acids, the present study was unable to investigate potential dose–response relationships. Nonetheless, it is important to note that the dose employed in our trial aligns with the American Heart Association’s recommendation for cardio-protection in individuals with a history of coronary disease. This recommended dose is at least twice the dosage suggested for cardiovascular protection in healthy populations, which is equivalent to consuming 1 to 2 servings of fish per week. In addition, we performed the subgroup analyses based on the dietary fish intake at baseline. Although analyses based on the plasma n–3 index at baseline would also have been of great interest, measurement of the plasma n–3 index at baseline was limited to 60% of the 25,871 participants, resulting in greatly reduced statistical power. Thus, we decided a priori to stratify the participants based on dietary fish intake at baseline.

## 5. Conclusions

Our win ratio analyses incorporating priority of individual components of a composite outcome showed that the marine n−3 fatty acid supplementation had suggestive but statistically nonsignificant beneficial effects for the primary hierarchical composite major CVD outcome in all participants; however, significant beneficial effects were found among the participants with lower dietary fish intake. These benefits principally came from preventing CHD events rather than stroke events. Thus, n−3 fatty acid supplementation appears to prevent fatal and non-fatal CHD events rather than stroke, especially in people whose usual dietary fish intake is relatively low. These results justify a future RCT to assess whether n–3 fatty acid supplementation decreases the risk of CVD events, focusing on people who eat little or no fish.

## Figures and Tables

**Table 1:** Baseline characteristics of the VITAL participants.

	n−3 Fatty Acids	Placebo
N	12,933	12,938
Age, mean (SD)	67.1 (7.1)	67.1 (7.1)
Female, n (%)	6547 (50.6)	6538 (50.5)
Race or ethnic group, N (%)		
Non-Hispanic white	9044 (71.5)	9002 (71.2)
African American	2549 (20.1)	2557 (20.2)
Non-African American Hispanic	491 (3.9)	522 (4.1)
Asian	200 (1.6)	188 (1.5)
Native American	120 (0.9)	108 (0.9)
Other or unknown	249 (2.0)	274 (2.2)
BMI, mean (SD)	28.1 (5.7)	28.1 (5.8)
Dietary fish intake, servings/week, median (IQR)	1.5 (0.9, 2.5)	1.5 (0.9, 2.5)
Current smoking, N (%)	920 (7.2)	915 (7.2)
Medication use for hypertension	6553 (51.0)	6678 (52.0)
Medication use for diabetes	1366 (10.6)	1374 (10.6)
Medication use lowering cholesterol	4800 (37.8)	4742 (37.3)

Abbreviations: VITAL, Vitamin D and Omega-3 Trial; SD, standard deviation; IQR, interquartile range; BMI, body mass index. The number of participants with available BMI data was 12,626 and 12,644 for the n−3 fatty acid and the placebo groups, respectively. The number of participants with available dietary fish intake data was 12,728 and 12,709 for the n−3 fatty acid and the placebo groups, respectively.

## Data Availability

Data described in the manuscript, codebook, and analytic code will be made available upon request pending application to J.E.M. and S.O.

## Supplementary Materials

The following supporting information can be downloaded at: https://www.mdpi.com/article/10.3390/nu15194235/s1, Table S1: The number of incident cases (%) for each component of the hierarchical composite outcomes in all participants of the VITAL study; Table S2: Results of win ratio analyses for the primary hierarchical composite outcome of major CVD events (prioritizing non-fatal stroke over non-fatal MI) and the secondary hierarchical composite outcome of expanded CVD events (the primary outcome + CABG/PCI, prioritizing non-fatal stroke over non-fatal MI) between n−3 fatty acid group and its placebo group in all participants of the VITAL study (*n* = 25,871); Table S3: Results of win ratio analyses for the primary hierarchical composite outcome of major CVD events (prioritizing non-fatal stroke over non-fatal MI) and the secondary hierarchical composite outcome of expanded CVD events (the primary outcome + CABG/PCI, prioritizing non-fatal stroke over non-fatal MI) between n−3 fatty acid group and its placebo group in subgroups with low and high fish consumption at baseline in the VITAL study.
