# Supplementary material for: Marine n−3 Fatty Acids and Prevention of Cardiovascular Disease: A Novel Analysis of the VITAL Trial Using Win Ratio and Hierarchical Composite Outcomes
Source: Nutrients. 2023 Sep 30;15(19):4235. doi: 10.3390/nu15194235 (PMC10574231; doi:10.3390/nu15194235)
Supplement: Supplementary file 1 [file nutrients-15-04235-s001.zip › nutrients-2634035-supplementary.pdf]

Supplementary Table S1. The number of incident cases (%) for each component of the hierarchical composite outcomes in all participants of the VITAL study

N (%)	N−3 fatty acids (N = 12,933)	Placebo (N = 12,938)
Fatal CHD	37 (0.29)	49 (0.38)
Fatal other CVD including stroke	105 (0.81)	99 (0.77)
Fatal MI	13 (0.1)	26 (0.2)
Fatal stroke	22 (0.17)	20 (0.15)
Non-fatal MI	132 (1.02)	174 (1.34)
Non-fatal stroke	126 (0.97)	122 (0.94)
Non-fatal CABG/PCI	244 (1.89)	283 (2.19)

Abbreviations: CVD, cardiovascular disease; CHD, coronary heart disease; MI, myocardial infarction; CABG, coronary artery bypass grafting; PCI, percutaneous coronary intervention.

Supplementary Table S2. Results of win ratio analyses for the primary hierarchical composite outcome of major CVD events (prioritizing non-fatal stroke over non-fatal MI) and the secondary hierarchical composite outcome of expanded CVD events (the primary outcome + CABG/PCI, prioritizing non-fatal stroke over non-fatal MI) between n−3 fatty acids group and its placebo group in all participants of the VITAL study (n= 25,871)

	The primary hierarchical composite outcome		The secondary hierarchical composite outcome	
Hierarchical priority	Winners	Losers	Winners	Losers
1 = Fatal CHD	600,424	453,196	600,424	453,196
2 = Fatal other CVD including stroke	1,159,082	1,222,934	1,159,082	1,222,934
3 = Non-fatal stroke	1,413,547	1,411,721	1,413,547	1,411,721
4 = Non-fatal MI	1,823,397	1,426,554	1,823,397	1,426,554
5 = Non-fatal CABG/PCI	NA	NA	1,749,502	1,660,783
Total	4,996,450	4,514,405	6,745,952	6,175,188
**Reciprocal win ratio[1] (95% CI)**	0.90 (0.79 to 1.04)	p = 0.16	0.92 (0.81 to 1.03)	p = 0.15

[1] Reciprocal win ratio < 1 means beneficial effect of n−3 fatty acids supplementation on the composite outcome.

Abbreviations: CVD, cardiovascular disease; CHD, coronary heart disease; MI, myocardial infarction; CABG, coronary artery bypass grafting; PCI, percutaneous coronary intervention; CI, confidence interval.

Supplementary Table S3. Results of win ratio analyses for the primary hierarchical composite outcome of major CVD events (prioritizing non-fatal stroke over non-fatal MI) and the secondary hierarchical composite outcome of expanded CVD events (the primary outcome + CABG/PCI, prioritizing non-fatal stroke over non-fatal MI) between n−3 fatty acids group and its placebo group in subgroups with low and high fish consumption at baseline in the VITAL-study

	Low fish intake (< 1.5 servings/week, n =13,515)	High fish intake (≥ 1.5 servings/week, n = 11,922)
	Reciprocal win ratio[1] (95% CI)	Reciprocal win ratio[1] (95% CI)
**The primary hierarchical composite outcome of major CVD events** (i.e., prioritizing fatal CHD, fatal other CVD including stroke, non-fatal stroke, and non-fatal MI in this order)	0.79 (0.65 to 0.97)	1.05 (0.86 to 1.30)
**The secondary hierarchical composite outcome of expand CVD events (the primary outcome + CABG/PCI)** (i.e., prioritizing fatal CHD, fatal other CVD including stroke, non-fatal stroke, non-fatal MI, and non-fatal CABG/PCI in this order)	0.85 (0.72 to 1.01)	1.01 (0.85 to 1.20)

[1] Reciprocal win ratio < 1 means beneficial effect of n−3 fatty acids supplementation on the composite outcome.

Abbreviations: CVD, cardiovascular disease; CHD, coronary heart disease; MI, myocardial infarction; CABG, coronary artery bypass grafting; PCI, percutaneous coronary intervention; CI, confidence interval.
